# Approach to the Patient With Prader–Willi Syndrome

**DOI:** 10.1210/clinem/dgac082

**Published:** 2022-02-12

**Authors:** Charlotte Höybye, Maithé Tauber

**Affiliations:** Department of Endocrinology, Karolinska University Hospital, StockholmSweden; Department of Molecular Medicine and Surgery, Karolinska Institute, Stockholm, Sweden; The Clinical and Scientific Advisory Board of the International Organization for Prader-Willi Syndrome, IPWSO; The Clinical and Scientific Advisory Board of the International Organization for Prader-Willi Syndrome, IPWSO; Centre de Référence Maladies Rares PRADORT (syndrome de PRADer-Willi et autres Obésités Rares avec Troubles du comportement alimentaire), Hôpital des Enfants, CHU Toulouse, Université Toulouse III, Toulouse, France; Institut Toulousain des Maladies Infectieuses et Inflammatoires (Infinity) INSERM UMR1291—CNRS UMR5051—Université Toulouse III, Toulouse, France

**Keywords:** Prader–Willi syndrome, children and adults, clinical characteristics, treatment

## Abstract

Prader–Willi syndrome (PWS) is a rare, multisystemic, genetic disorder involving the hypothalamus. It is caused by loss of expression of paternally inherited genes in chromosome 15 q11-13 region. The estimated incidence is around 1 in 20.000 births. PWS is characterized by a complex lifelong trajectory involving neurodevelopmental, nutritional, endocrine, metabolic, and behavioral changes. The major symptoms are hypotonia, short stature, hypogonadism, and eating disorders ranging from anorexia in infancy to hyperphagia, a deficit of satiety, and a high risk of severe obesity. The patients display intellectual disability comprising cognitive deficit, delayed motor and language development, learning deficits, impaired social skills, and emotional regulation. Behavioral features including temper outbursts, anxiety, obsessive–compulsive symptoms and rigidity are common and become more apparent with increasing age. Almost all have hypogonadism and growth hormone deficiency. Central adrenal insufficiency is rare whereas central hypothyroidism occurs in up to 30% of children with PWS. The prevalence of obesity increases with age from almost none in early childhood to more than 90% in adulthood. Up to 25% of adults with obesity have type 2 diabetes. Obesity and its complications are the major causes of comorbidity and mortality in PWS. As there is no specific treatment, care consists of comprehensive management of feeding disorders, a restricted, controlled diet, regular exercise, hormone substitution, and screening and treatment of comorbidities. Here we present the course of PWS from birth to adulthood in 2 patients and discuss their symptoms in relation to the literature.

Prader–Willi syndrome (PWS) is a rare, multisystemic, genetic, neurodevelopmental disorder characterized by impaired hypothalamus development and function (1). The incidence of PWS is approximately 1 in 16.000 to 1 in 21.000 live births (2, 3) and PWS is caused by loss of expression of paternally inherited genes in the chromosome 15 q11-13 region (1, 4). PWS was for the first time described 1956 by Prader et al (5), and since then the knowledge of the syndrome has expanded. PWS is characterized by a complex trajectory involving neurodevelopmental, nutritional, endocrine, metabolic, and behavioral changes throughout life. The major symptoms of the syndrome in childhood are hypotonia, poor growth, and short stature. Feeding problems from anorexia at birth switching to excessive weight gain, hyperphagia, and satiety deficits often result in morbid obesity (6). In addition, the patients with PWS display intellectual disability (ID) and behavioral problems (1). The symptoms of PWS are highly variable between individuals and with age. Early diagnosis of PWS is important for initiating correct and timely comprehensive management. Here we present 2 patients with PWS and discuss their symptoms in relation to the literature. The clinical presentations have been modified to avoid identification.

## Case 1

The patient is a 28-year-old man with PWS. He was born at term by Cesarean section. At birth he weighed 2001 g, length was 50 cm, and he had nondescended testes. Due to typical symptoms of PWS a genetic analysis was performed at the age of 2 months and it showed paternal deletion. The first year of life was complicated by hypotonia with feeding difficulties and a weak cry. At the age of 2 his appetite increased, and weight increased at a normal rate. At the age of 5 he developed hyperphagia but, fortunately, his family proved resourceful and invested a lot of time and effort in following the recommendations given by the pediatric multidisciplinary team and his body mass index (BMI) remained in the normal to slightly overweight range.

His cognitive and motor developments were delayed, and he went to a special educational class. Growth was insufficient. Growth hormone (GH) stimulation test showed GH deficiency and he started on GH treatment (0.033 mg/kg/day) at age of 4 years. He managed injections himself (under supervision) and hardly missed any of them. During the following years he grew 6 to 12 cm/year. Weight was stable (±0 SD) and no adverse effect occurred. At the age of 7 the patient was diagnosed with scoliosis, which was subsequently treated with bracing. At the age of 11 years, the patient underwent bilateral orchidopexy. The operation was uncomplicated, while the postoperative period was complicated by aggressive behavior and temper outbursts. At the age of 13 years, he developed pubertal signs: pubic hair scored 3 and genital development 2 on the Tanner scale. Testicular volume was 3 mL.

At 16 years, growth velocity decreased to 2.2 cm/year and the GH dose was increased. The scoliosis worsened and was corrected with a fixation operation. The operation went well, but again the patient got aggressive postoperatively. During the following years, the patient’s mood fluctuated. He refused blood tests to be taken and he requested to manage the GH injections himself without help. A transition meeting between the pediatrician and the adult endocrinologist was organized when the patient was 18 years old. He had reached his adult height, and it was agreed to perform a GH stimulation test before he was referred to the adult endocrinologist. At the age of 19 years, the patient came for the first visit in the adult department. The patient was feeling fine. He was in the last year of “high school” and was living with his parents. His height was 172 cm, weight 64.6 kg, BMI 21.6 kg/m^2^. Fat mass was 16 kg (24%). He was prescribed 1.4 mg GH/day that he injected himself unsupervised. Insulin-like growth factor I (IGF-I) was 630 µg/L (160-420). Fasting glucose and HbA1C were normal. GH treatment was discontinued, and after 2 months, an insulin tolerance test was performed. Peak GH was 2.97 µg/L (glucose nadir 1.7 mmol/L [31 mg/dL]). IGF-I was 86 µg/L (–3.2 SDS) and the patient restarted GH treatment. He was routinely followed by the endocrinologist and the patient was in a stable physical condition. However, he had large fluctuations in his mood, for which he was seeing a psychiatrist. At 24 years of age, the patient moved to a group home and got a job in a day care for dogs, where he is still working. He is on a controlled, strict diet, and besides the exercise he gets by working he also swims and dance. Weight is stable. He is on a GH dose of 0.4 mg/day that he is injecting under supervision. At latest visit weight was 69.5 kg, BMI 23.2 kg/m^2^. IGF-I normal (298 µg/L [150-390]). Testosterone low (5.4 nmol/L [10-30]), luteinizing hormone 1.9 U/L, follicle-stimulating hormone 7.6 U/L. HbA1C 3.9% (3.6-5.0 (Mono-S method42). Total cholesterol 4.6 mmol/L, high-density lipoprotein cholesterol 1.4 mmol/L, low-density lipoprotein cholesterol 3.2 mmol/L. Replacement of the hypogonadism has been discussed several times, but the patient refuses. He is most of the time pleased with life but has recurring periods of bad mood and temper outbursts.

## Case 2

The patient is a 40-year-old woman with PWS. The initial diagnosis of PWS was based on clinical criteria, and a DNA methylation analysis at the age of 4 confirmed it. During pregnancy, the patient’s mother noticed decreased fetal movements. Pregnancy was otherwise normal. Delivery was at term and without complications. Birth length and weight could not be retrieved but were reported to be normal. She had a severe hypotonia and difficulties to breast feed and was fed using a widened fast-flow nipple. Weight and length increased slowly. During early childhood appetite increased. Psychomotor development was delayed but she managed education in an ordinary school with extra help. The family had a well-established contact with the hospital’s pediatric team and followed their recommendations, except for a restricted, controlled diet. The patient was participating in several physical activities and did not become obese. However, the increase in height was less than expected.

When the patient was 10 years old, she was enrolled in one of the first studies of GH treatment in children with PWS. After GH treatment was initiated, a clear increase in height was seen and the tolerance of the treatment was good. After 5 years on GH, the patient started to gain further in weight. The increase in weight continued (BMI 30.8 kg/m^2^) and she developed type 2 diabetes at 15 years of age. GH treatment was discontinued, and antidiabetic treatment initiated. During the following 2 years, the patient lost weight, blood glucose decreased, and GH treatment was restarted. After GH treatment was resumed, the patient increased a few centimeters in height. At completion of growth, height was 163.7 cm and weight 78.5 kg. She complained of back pain, but an X-ray did not show scoliosis nor kyphosis. At 19 years of age, a transition meeting between the pediatrician and the adult endocrinologist was arranged and it was agreed to perform a GH stimulation test (insulin hypoglycemia test) before transferal to the adult clinic. The test showed peak GH values fulfilling the criteria for adult GH deficiency (GH peak 2.5 µg/L) and she resumed GH treatment with 1.2 mg GH/day. When she came for the first visit in the adult clinic, she was feeling fine, and she was in the last year of “high school.” Blood glucose and IGF-I (260 µg/L) were normal. Pubertal development was delayed: breast development Tanner stage 4 but no menarche. The patient again gained weight. At 24 years of age, BMI was 32.7 kg/m^2^. Fasting blood glucose was elevated, HbA1C was 7.0%, and insulin treatment was initiated. The patient was feeling fine, and she and her parents wanted to continue GH treatment. After she had finished school, the patient got sheltered work where she was packing different things and she was living partly in her family home and partly in a small non-PWS–specialized residential home. Food was not restricted at work or at the residential home. The patient was referred to the diabetes team in the hospital. Examinations did not show any complications of the diabetes. She continued with GH treatment. During the following years, her weight continued to increase. Metabolic control was acceptable for a long time (HbA1C 6.5%), but at the age of 34 years the patient’s weight was 120 kg (BMI 43 kg/m^2^) and HbA1C had increased to 8.5%, and consequently GH treatment was discontinued. Glucagon-like peptide-1 (GLP-1) analog treatment was started, and her weight decreased 3 kg and her HbA1C to 7%. The patient developed sleep apneas treated with continuous positive airway pressure. Two years ago, she moved to a specialized PWS group home and has lost 30 kg. Antidiabetic treatment has been reduced, and insulin treatment discontinued.

## Clinical Symptoms and Signs of PWS

PWS is a multi-organ syndrome, and some symptoms are more distinct in some patients than in others (1). Clinical characteristics and trajectory of PWS are typical in the 2 patient reports. The major characteristics of PWS are displayed in Table 1. The classical symptoms include dysmorphic features with a long narrow face, small hands and feet, and narrow hands with a straight ulnar border. In the neonatal period, severe hypotonia, poor sucking, and failure to thrive are prominent. During infancy and early childhood, hypotonia improves but remains present at all ages. A mild to moderate cognitive deficit and delayed psychomotor development become increasingly apparent along with impaired skeletal growth.

**Table 1.** Prader–Willi syndrome in brief

**Epidemiology**
Incidence 1/20.000 births
**Etiology**
Lack of expression of paternally inherited genes in chromosome 15 q11-13 region (65-70% paternal deletion, 30-35% maternal disomy, 1-5% imprinting defects)
**Major symptoms and signs**
Infants
Hypotonia
Feeding difficulties
Developmental delays
Genital hypoplasia
Growth hormone deficiency
Childhood
Hypotonia
Developmental delays
Intellectual disabilities
Behavioral problems, compulsive and ritualistic behaviors, autism spectrum disorder, emotional lability, temper outbursts, poor social skills
Growth hormone deficiency and short stature
Genital hypoplasia, hypogonadism, absent or incomplete puberty
Hyperphagia and risk of severe obesity
Adults
Intellectual disabilities
Behavioral problems as above
Hyperphagia and risk of severe obesity
Increased risk of comorbidities
Hypogonadism
Growth hormone deficiency
Other symptoms
Scoliosis
Sleep problems, sleep apnea
Decreased bone mineral density
Skin picking

Hyperphagia usually starts at the age of 4 years and requires a restricted and constantly supervised diet with strict control of food access by parents and caregivers as well as scheduled physical activities to prevent early development of morbid obesity (6). The frequency of obesity in PWS varies from 40% in children to 82% to 98% in adults (1, 7). Independent of the degree of obesity, body composition is abnormal in PWS with more body fat than lean body mass; the use of BMI mitigates evaluation of the adiposity (8, 9). Scoliosis is very frequent, up to 80% at adolescence, with classically 2 peaks of occurrence: 1 before 4 years of age for early-onset scoliosis and 1 at adolescence close to that of idiopathic adolescent scoliosis but occurring with the same frequency in both sexes (1). Central and obstructive sleep apnea (OSA) and excessive daytime sleepiness with or without narcolepsy are common (1). Cataplexy is also frequently reported in children (1).

Cryptorchidism in boys (close to 100%) and genital hypoplasia related to hypogonadism are very frequent (10). In both genders, puberty is delayed and incomplete leading to a decreased or absent growth spurt and consequently reduced adult height if not treated (1, 11). The incidence of premature adrenarche is around 30% and it may lead to early start of puberty and incomplete and accelerated bone maturation with decreased growth and adult height if unrecognized and not treated. Fertility in PWS is not known in detail. Pregnancies have been reported in females in publications and reports at scientific meetings but no male with PWS has fathered a child.

Patients with PWS have behavioral characteristics including temper tantrums, a stubborn and controlling behavior, compulsive–obsessive symptoms, deficits of emotional regulation, and rigidity (12). Psychiatric and behavioral problems become more prominent in adolescence and adulthood, and together with the patients’ hyperphagia and cognitive disabilities they are the main limitations to independent living.

A recent study of 70 adults with PWS showed that 61% had undiagnosed health problems (13). Seventy-four percent had scoliosis, 18% hypertension, 19% hypercholesterolemia, 17% type 2 diabetes mellitus, and 17% hypothyroidism (13). Mortality rates of 0.25% to 3% per year have been reported (14-18). Many deaths are sudden and unexpected, and they occur across the lifespan (18, 19). Major causes of death in younger children are respiratory infections; in adolescents and young adults accidents, gastrointestinal perforation, and choking (related to dysphagia and hyperphagia); and in adults complications of obesity, including cardiac and respiratory failures, and thromboembolic problems (14-19).

## Genetics in PWS

When suspected by clinical findings, usually identified in infancy, the diagnosis is confirmed by genetic testing. A DNA methylation analysis detects >99% of affected individuals and fluorescence in situ hybridization, methylation-sensitive methylation ligation–dependent probe amplification or chromosomal microarray further characterize molecular class (4). Genetic subtype analyses are important for counseling about symptoms and recurrence risk. The genetic mechanisms include paternal 15q microdeletion (65-70% of cases), which can be further divided into Type I and Type II, or atypical deletions, chromosome 15 maternal uniparental disomy (30-35% of cases) and imprinting defects (1-5% of cases) (4). In neonates the deletion/nondeletion ratio is now around 1:1. PWS is usually caused by a spontaneous mutation (4). The likelihood for recurrence is estimated to be 1%, but in rare cases with unbalanced translocations or imprinting defects the risk of recurrence can be up to 50% (4).

## Endocrinology in PWS

PWS is a hypothalamic disease, but only a few histopathological examinations of the hypothalamus have been performed (20). They have shown abnormalities in the hypothalamus, in particular low content of oxytocin neurons (20), and magnetic resonance imaging studies have shown abnormalities of the pituitary (hypoplasia, empty sella) in 63-74% (21).

Although there is no precise understanding of the entire hypothalamic disorder, PWS mice models including Magel2 and Snord116 gene–inactivated mice (22-25) showed abnormal oxytocin-secreting neurons, development and function, and a defect of hormone maturation via decreased levels of Proconvertase 1 enzyme in the hypothalamus, pancreas, and stomach, explaining various levels of deficits of matured hormones (26). More recently it has been shown that the PWS phenotype is associated with a wide range of epigenetic modifications, not only in the PWS chromosomal region. The modifications are mostly observed in brain development, and endocrine and endocrine resistance pathways (26).

Children with PWS have reduced GH responses to several different GH secretion stimulation tests as well as decreased spontaneous GH secretion in 58% to 100% (27). Since 2000, GH treatment in children with genetically confirmed PWS without prior GH stimulation testing has been approved in many countries (1, 27). In contrast, GH treatment in the transition period and in adults with PWS without prior GH stimulation testing is only approved in a few countries (The Netherlands and Poland), and in most countries reassessment of GH secretion must be performed and results evaluated according to guidelines for the diagnosis of GH deficiency in adults (28). Most GH-stimulating tests measure the secretion of GH from the pituitary, which can be falsely normal in patients with PWS due to the hypothalamic origin of GH deficiency (1, 27). In addition, the increased amount of adipose tissue and hypogonadism might confound the results in adults. In studies reporting results from GH testing in adults, profound GH deficiency, defined according to existing diagnostic criteria, was demonstrated in 0 to more than 50% (27, 29). IGF-I levels are affected by obesity and sex steroid deficiency, and might be difficult to evaluate, but most patients with PWS have low levels of IGF-I (27, 30-32).

The effects of GH treatment in children with PWS are well documented (27). GH treatment in children with PWS improves height, head circumference, facial appearance, bone strength, increases lean body mass, and reduces fat mass (27). In addition, it has beneficial effects on blood lipids and improves cognition and quality of life. In a recent placebo-controlled GH crossover trial, it was shown that in GH-treated young adults with PWS who had attained adult height, fat mass increased during placebo treatment, whereas fat mass decreased and lean body mass increased when resuming GH treatment (32). Nowadays, treatment with GH is initiated in early infancy usually between 3 and 9 months of age, and has dramatically changed the clinical status of patients with PWS. GH (27). For both children and adults, IGF-I level is usually in the upper range of normal for appropriate reference ranges even with low doses of GH (27). IGFBP-7, an IGF binding protein involved in the regulation of the binding to the IGF-I receptor, was recently shown to be elevated in children with PWS and normalized on GH treatment indicating that IGFBP-7 may be a marker of GH sensitivity and that higher levels of IGF-I are acceptable in PWS (26).

A recent meta-analysis of GH treatment of adults with PWS, including 9 randomized controlled trials and 20 nonrandomized controlled trials, showed that body composition improved during 12 months of GH treatment with an increase in mean (95% CI) lean body mass of 1.95 kg (0.04-3.87 kg) and a reduction of mean (95% CI) fat mass of –2.23% (–4.10% to –0.36%) (33). BMI, low-density lipoprotein cholesterol levels, fasting glucose levels, and bone mineral density were unchanged. Improvements in physical activity has been shown in open label studies using treadmill, accelerometers, and measurements of peak expiratory flow and quality-of-life (QoL) questionnaires (1, 27). Evaluation of QoL in adults with PWS is difficult because of the intellectual disability, and the behavioral and psychiatric problems, and only a few studies have focused on this outcome. However, improvements in QoL (less optimistic by the caregivers/relatives than by the patients) was reported in all 4 studies and in neuropsychological tests in another study (1, 27). GH treatment for 2 to 3 years did not increase bone mineral density, probably because of the relatively short period of treatment (27, 32). On the other hand, bone formation markers increased, whereas resorption markers did not change. These findings indicate that low bone mineral density is not only related to impaired GH secretion.

Reported side effects to GH treatment in PWS are few and no major side effects have been observed (27, 32-35). The number of patients who developed scoliosis or diabetes did not increase, but uncontrolled diabetes, complications to diabetes, and active cancer are contraindications to GH treatment in PWS (27, 33-35). At initiation, GH treatment might lead to lymphoid enlargement and as central and obstructive apnea are common, polysomnography should be performed before starting GH therapy in children (27). In a study on GH treatment for 3 years in adults with PWS, sleep apnea did not increase (36). Theoretically, there might be a risk for development of central adrenal insufficiency and central hypothyroidism during GH treatment, and in the presence of a clinical suspicion these should be ruled out. In addition, GH treatment is probably not relevant in patients with uncontrolled severe obesity.

The majority of patients with PWS are hypogonadal (10). A combined central and peripheral form of hypogonadism is often present in PWS, but the degree of hypogonadism is variable and less pronounced in women (10, 11). In both genders, puberty is not completed, and sexual maturation stops at the early to mid-puberty stages, less clearly in girls as development and maturation of the breasts are almost complete, whereas spontaneous menarche is rare and delayed (1, 11). Induction of puberty is needed for skeletal growth, maturation of muscles and bone, as well as development of an adult appearance (11). For many years there has been a hesitating attitude toward treatment with sex steroids: in males, partly because of a worry of inducing aggressiveness; in females, partly because the production of estrogen in fat cells is anticipated to be sufficient, but also because of the risk of thromboembolic events and breast cancer and an uncertainty about the patients’ management of menstrual bleedings. There is only 1 observational study of testosterone treatment of males with PWS, showing improved secondary sex characteristics and body composition (37). No systematic trials on sex steroid treatment have been published for women with PWS. However, over time the confidence in and the acknowledgement of the importance of sex steroid treatment has increased, and the treatment has become more common. Treatment with sex steroids should preferably be introduced during puberty and continued in adult life but potential benefits and risks must be balanced, and it is important to understand the distinction between treatment of hypogonadism and birth control management.

Despite the high frequency of hypogonadism, in clinical experience many adults with PWS have strong romantic thoughts and an interest in sexual experiences. Studies have shown that some women with PWS have low normal inhibin-B levels, potentially indicating fertility (1, 11). Reports of 11 pregnancies in 6 PWS women have been published worldwide; in 2 women the PWS diagnosis was based on a clinical diagnosis, 3 had a paternal deletion, and 1 uniparental disomy (38, 39). Fertility in PWS includes medical and ethical considerations, and appropriate anticipatory guidance, counseling, and education is important. Information about the 11 pregnancies in women with PWS indicate that the gestations were uncomplicated, 7 babies were born vaginally, 3 were delivered by planned caesarean sections and 1 with emergency cesarian section (38, 39). The mothers were unable to breast feed and the infants were mostly taken care of by others. Four of the 11 children were healthy.

The function of the hypothalamus–pituitary–adrenal axis was believed to be normal in PWS until 2008, when a study showed insufficient adrenocorticotropin response to a metyrapone test in 15 out of 25 (60%) children with PWS (40). It has therefore been speculated whether undetected central adrenal insufficiency could be the cause of sudden deaths, particularly deaths occurring during infections and stressful events (41). However, subsequent studies in children and adults could not confirm the high prevalence of CAI and found rates of CAI ranging from 0 to 14% (41, 42). Some degree of CAI may be part of PWS, but clinically relevant hypocortisolism appears to be rare (42).

In a large study of 339 patients with PWS aged 0.2 to 50 years, hypothyroidism secondary to hypothalamic failure was reported with a high frequency in children and adolescents (7.8%) but less frequent in adults (4.2%) (43). Corresponding rates for primary hypothyroidism were 1.7% and 2.1%, respectively (43). Interestingly, a very high prevalence (72.2%) of central hypothyroidism was reported in infants with PWS (44), and another investigation of 27 individuals with PWS (age 3 months to 39 years) showed central hypothyroidism in 19% (45). Reports on triiodothyronine (T3) levels in PWS are limited (46), but data for 142 children with PWS showed normal values in a great majority (47). Peripheral conversion of free thyroxine (T4) to T3 might explain the relatively low free T4 levels detected in PWS which can be augmented during GH treatment. Anyhow, the significant presence of central hypothyroidism confirms the hypothalamic–pituitary dysfunction in PWS, and as some of the symptoms of hypothyroidism are similar to some of the symptoms of PWS regular monitoring of thyrotropin, free T4, and free T3 are required and when indicated doses of levothyroxine should be administered.

The regulation of thirst is anticipated to be normal in PWS, whereas hyponatremia and SIADH (syndrome of inappropriate antidiuretic hormone or vasopressin secretion) occurs. In a recent international study of 1326 patients (68% adults), 34 (2.6%) had at least 1 episode of mild or moderate hyponatremia (125 ≤ Na < 135 mmol/L) (48). The causes included psychotropic medication (32%), excessive fluid intake (24%) and hyperglycemia (12%). Seven (0.5%) adults had severe hyponatremia (Na < 125 mmol/L), and 2 died. Two patients were on desmopressin for nocturnal enuresis, 2 had excessive fluid intake, 1 adrenal insufficiency, 1 was on diuretic treatment and in 1 the cause was unknown. Thus, hyponatremia is rare but potentially fatal.

## Hyperphagia and Obesity

Hyperphagia is a major problem in PWS and is observed in all genetic subgroups, although more frequent in patients with deletion. Hyperphagia negatively affects QoL, social life, school, and work and requires a lifelong restricted and controlled diet. It has a significant impact on the person with PWS, the caregivers and the entire family (49). The description of the 6 nutritional phases in PWS has greatly improved the understanding of the natural history of PWS (6). The 6 postnatal nutritional phases range from poor feeding and failure to thrive (Phase 1a), to normal eating without (Phase 1b) and then with excessive weight gain (Phase 2a), to an increased interest for food with weight gain (Phase 2b) followed by occurrence of hyperphagia and high risk of life-threatening obesity (Phase 3), and in some older adults reduced appetite and improved satiety (Phase 4) (6).

The etiology of hyperphagia is not completely mapped (1, 7). Regulation of appetite and satiety is complex and involve a multitude of peptides. Various peptides regulate appetite with hormones that suppress appetite, *anorexigenic* hormones, which are leptin, cholecystokinin, peptide YY, pancreatic polypeptide, GLP-1, obestatin, oxytocin, and brain-derived neurotrophic factor, and insulin and hormones that promote appetite, *orexigenic* hormones, which are ghrelin, neuropeptide Y, Agouti-related protein, and hypocretin (orexin) (7).

Several clinical trials for the treatment of hyperphagia have been performed or are ongoing. Oxytocin promotes satiety but clinical trials with oxytocin could not demonstrate an effect on food-related behavior whereas in a phase 2 cohort study a positive effect on feeding and social skills was seen in infants with PWS (1, 50). Another peptide that has been extensively studied is ghrelin, which is secreted in the stomach. Hyperghrelinemia is seen in all ages in patients with PWS (51) and is suggested to be involved in the increased appetite in PWS. Circulating ghrelin is present in an acylated form, which increases appetite (the hunger hormone), and an unacylated form, which decreases appetite antagonizing the effect of the acylated form (1, 51). An intrinsic defect in the ratio between the 2 forms has been suggested with a relative acylated deficit in infants and an excess later correlating with the change in eating behavior (1, 51). Although short studies with an unacylated ghrelin analog showed changes in food behavior (52), longer duration studies were not able to demonstrate a significant improvement in food-related behavior and reduced hunger. Several other clinical trials on different drugs affecting hyperphagia are ongoing, among them trials with GLP-1 agonists, diaxoxide, carbetocine, and serotonin–noradrenalin–dopamine reuptake inhibitors and possibly others.

## Insulin and Diabetes

Although, obesity is frequently present, several studies have reported normal insulin levels and higher insulin sensitivity in PWS than in BMI-matched children and adults (8, 53-57). Increased insulin sensitivity has been interpreted to be caused by less visceral compared with subcutaneous fat tissue, high adiponectin, and ghrelin levels (8, 56), but low levels of GH might also play a role together with a degree of insulinopenia and a decreased plasma ratio insulin/proinsulin (55). Other studies have shown decreased insulin response to glucose stimulation (53, 54), while 1 study showed decreased insulin secretion in children with PWS (57).

The frequency of type 2 diabetes is reported to be 0 to 25% depending on the study population (34, 47, 58-60). A recent study showed that diabetes in young, obese adults was diagnosed at a mean age of 16 years with a peak during the transition period, suggesting reinforced glucose monitoring in adolescents and young adults (61). Insulin resistance and obesity are well known risk factors for type 2 diabetes and higher insulin levels and a high homeostatic model assessment index has been demonstrated in obese compared with nonobese adults with PWS (34, 59, 60). Likewise, an increase in fasting insulin and insulin resistance was observed with increasing age and BMI in children with PWS (56). Like other individuals, heredity for type 2 diabetes increases the risk for this disease, and diagnosing and monitoring of diabetes type 2 should follow general guidelines.

## Sleep Abnormalities

Respiratory sleep abnormalities are well documented in PWS and include both central and obstructive apnea (62). Over time, untreated OSA may lead to cardiovascular complications including hypertension, cor pulmonale, and stroke (63). In addition to long-term adverse cardiovascular and respiratory events associated with untreated OSA, sleep interrupted by respiratory events and arousals may cause daytime sleepiness, compromise cognitive function, worsen executive function and memory, and increase behavior problems in people with PWS. The complications might be related to hypoxemia and sleep fragmentation (64). Prevention of obesity is important to minimize the risk of developing OSA, as well as to keep metabolic parameters and blood pressure normal. OSA is treated with continuous positive airway pressure and tonsillar hypertrophy with tonsillectomy (36, 62, 64).

Patients with PWS also display excessive daytime sleepiness and different forms of hypersomnia disorders, including narcolepsy with or without cataplexy, and it is important to investigate their sleep carefully and regularly in collaboration with a sleep expert team.

## The Future

As shown in the 2 case reports, PWS is an evolving disease characterized by a developmental trajectory including nutritional, endocrine, and metabolic, behavioral, and neurodevelopmental dimensions. Diagnosis at a very early age allows implementation of adequate multidisciplinary strategies to optimize care and successfully prevent obesity, although not all benefit from this comprehensive approach. Early treatment with oxytocin in infants with PWS has promising results by improving feeding and social skills (50), and may change the course of the disease but must be confirmed. An ongoing international study will answer this question. In adults, results from studies on comorbidities, most often related to obesity, are important for early prevention and treatment. In addition, more studies on the PWS behavioral phenotype and treatment of it are awaited.

Robust (randomized placebo controlled) studies showing reduced cardiovascular morbidity and mortality in adults with PWS treated with GH have not been performed. For such studies, a very large number of patients with PWS would be needed over many years and given the incidence and prevalence of PWS it is unlikely that they will be performed, and until they are we have to rely on observational data. There is only 1 observational study on long-term GH treatment of adults with PWS (65). In this study, body composition was shown to be normal in 22 adults with PWS treated with GH for a median time of 20 years, although the lipolytic and/or anabolic effect was less pronounced in women (65). No serious side effects were observed. Thus, treatment with GH has the potential to relieve some of the adverse effects of PWS, but it is an expensive treatment.

PWS is a model for approaching and understanding severe obesity with a hypothalamic origin. We hope that new or modified molecules used in ongoing or future clinical trials will offer a real possibility to cure or significantly mitigate hyperphagia and behavior issues in the near future. Patients with PWS are not a homogeneous group of patients, and future studies of lager cohorts with the potential to study subgroups and define individualized treatment are warranted.

## Conclusion

PWS is a multisymptomatic and complex disorder affecting the hypothalamic–pituitary function, resulting in several endocrine disorders. It also affects mental health and includes cognitive deficit, hypotonia, and hyperphagia, and a high risk of obesity. Early diagnosis is key for timely initiation of GH treatment, a restricted, controlled diet, regular physical activity, and initiation of efforts on mental health and development. In adulthood, these treatments continue but several comorbidities, especially related to obesity, are often present, and it is important to be aware that the physical and cognitive consequences of the syndrome might result in a complex and different clinical status. Pediatric and adult endocrinologists in cooperation with other specialists have a crucial role in the care of patients with PWS. Over time the health of children and adults with PWS has improved and the treatment options increased. The knowledge about PWS continues to increase and several studies are ongoing related to physical and mental issues.

## Data Availability

Data sharing is not applicable to this article as no datasets were generated or analyzed during the current study.

## Abbreviations

BMI: body mass index
GH: growth hormone
GLP-1: glucagon-like peptide-1
ID: intellectual disability
IGF-I: insulin-like growth factor I
IGFBP: insulin-like growth factor I binding protein
OSA: obstructive sleep apnea
PWS: Prader–Willi
QoL: quality-of-life
T3: triiodothyronine
T4: thyroxine
